# Characteristics of HIV-1 Natural Drug Resistance-Associated Mutations in Former Paid Blood Donors in Henan Province, China

**DOI:** 10.1371/journal.pone.0089291

**Published:** 2014-02-19

**Authors:** Yi Bao, Di Tian, Ying-Ying Zheng, Hong-Li Xi, Dan Liu, Min Yu, Xiao-Yuan Xu

**Affiliations:** Department of Infectious Diseases, Peking University First Hospital, Beijing, China; University of Cincinnati College of Medicine, United States of America

## Abstract

**Background:**

Natural drug resistance is a major cause of antiviral treatment failure. The characteristics of HIV-1 natural drug resistance-associated mutations in former paid blood donors in Henan Province remain unclear.

**Methods:**

One hundred and fifty HIV-1-positive plasma samples were collected. Plasma viral RNA was extracted for pol gene amplification and sequencing. The sequencing results were submitted to the HIV-1 drug resistance database for drug-resistance analysis.

**Results:**

The rates of natural drug resistance and resistance-associated mutations were 17.7% (19/107) and 40.2% (43/107), respectively. The rates of PI major, PI minor, NRTI, and NNRTI mutations were: 0, 30.8% (33/107), 10.3% (11/107), and 18.7% (20/107), respectively. Nine cases (8.4%) had both NRTI and NNRTI resistance-associated mutations. Seven cases (6.5%) had PI minor, NRTI and NNRTI resistance-associated mutations. NNRTI resistance was the most serious, followed by NRTI resistance and PI resistance. Polymorphism mutation sites with mutation rates in the protease region higher than 60.0% were: L63A/P/S/T 89.7%, V77I 82.2%, I72E/M/K/T/V 80.4%, I93L 75.7%, and E35D 72.9%. Polymorphism mutation sites with mutation rates in the RT region higher than 60.0% were: I135A/L/M/R/T/V 93.5%, T200A/E/I/P/V 89.7%, Q278E/K/N/T 88.8%, S162C/Y 82.2%, and K277R/S 66.4%. The distribution of 107 gene sequences was scattered, with some drug-resistant strains grouped in the same cluster.

**Conclusion:**

The natural drug resistance mutation rate of HIV-1 in former paid blood donors in Henan Province was 17.7%, with NNRTI resistance the most serious. The distribution of drug-resistant strains was scattered, with some correlations found in certain resistance loci.

## Introduction

Under natural circumstances, HIV can destroy the human immune system 10–15 years after the development of AIDS. At this stage, the patients may display a variety of opportunistic infections, tumors or other serious clinical symptoms [1]–[3]. Up to now, there are no effective drugs and therapies for complete cure of AIDS. Extensive application of ART has greatly reduced the morbidity and mortality of HIV/AIDS patients [4]–[6].

According to the AIDS epidemic report from Joint United Nations Programme on HIV/AIDS(UNAIDS) and the World Health Organization (WHO) [7], there were 34 million HIV carriers and AIDS patients, 2.7 million new HIV infections and 1.8 million deaths worldwide in 2010. By the end of 2011, in low-and middle-income countries, a total of more than 8 million HIV carriers received antiretroviral therapy, of which about 562,000 were children. During 2003–2011, the number of patients receiving antiretroviral therapy increased by 20 times in developing countries. During 2010–2011, the number of patients needing antiretroviral treatment increased by 20%. Based on the mechanism of action, antiviral drugs are divided into different groups, which include protease inhibitors, reverse transcriptase inhibitors, integrase inhibitors, fusion inhibitors, entry inhibitors and mixture of different agents. Among them, protease inhibitors and reverse transcriptase inhibitors are used more often in clinics[8]–[11].

However, HIV genome replication is error-prone. The virus exists in patients as a large and complex pool of quasispecies form [12], [13]. The selection pressure of antiviral drugs causes the accumulation of resistant strains that gradually become the dominant strains, leading to resistance to protease inhibitors and reverse transcriptase inhibitors. In addition, a resistance mutation caused by one drug may confer resistance to another drug that is not used, a phenomenon called cross-resistance[14]–[17]. Numerous studies have shown that drug resistance mutation is one of the main reasons leading to clinical antiviral treatment failure. Furthermore studies in the United States and Western Europe showed that the spread of drug-resistant strains increased significantly, and about 10.7% ∼ 27.6% of new infections were caused by drug-resistant strains of HIV-1[18]–[19]. Natural drug resistance is a major cause of antiviral treatment failure. Before antiviral treatment, drug resistance tests for HIV-1 strains can guide the clinical medication, which will greatly reduce the risk of initial treatment failure[20]–[25].

Since 2003, most patients undergoing AIDS antiretroviral treatment were former paid blood donors in Henan Province. HIV drug resistance caused by antiretroviral therapy has become a major obstacle to clinical treatment. In recent years, drug resistance data in Henan Province have been reported[26]–[27], but the research about primary drug resistance is lacking. With infection rates rapidly rising, the primary drug resistance data also need to be constantly updated and supplemented.

In this study, we investigated the characteristics of HIV-1 natural resistance mutations in former paid blood donors in Henan Province. Understanding primary drug resistance before antiretroviral treatment may facilitate the development of effective treatment programs in order to achieve the greatest degree of viral suppression, and understand molecular evolution traceability rules of HIV-l resistance genes. It is also important for reducing the prevalence of drug-resistant strains and the effective use of drugs.

## Methods

### Ethics Statement

This study was approved by the Ethical Committee of Beijing You’an Hospital Affiliated to Capital Medical University and written informed consent was obtained from all participants. Each consented participant was given a unique number. The biological and behavioural information was linked anonymously to protect the participants’ privacy. This procedure was approved by the ethics committee.

### Samples

Between 2005 and 2007, 150 plasma samples were collected in Beijing YouAn Hospital from paid blood donors in Henan Province. All patients were confirmed as HIV-1 positive using Western blot. The ages of patients were between 28 and 60 years, with a mean age of 44.7 years. There were 86 male cases, accounting for 57.3%, and 64 female cases, accounting for 42.7%. HIV infected peripheral blood samples were collected using EDTA anticoagulant tubes before treatment with antiviral drugs. Plasma samples were stored at −80°C.

### Nucleic Acid Extraction

HIV-1 RNA was extracted from 200 ul of plasma using the MiniBEST Viral RNA/DNA Extraction Kit (TaKaRa, Dalian, China) according to the manufacturer’s instructions.

### Nested Polymerase Chain Reaction

HIV-1 Pol gene was amplified using nested-PCR. The TaKaRa PrimeScript One Step RNA RT-PCR Kit(TaKaRa, Dalian, China) was used for the first round of PCR. TaKaRa Ex Taq Kit(TaKaRa, Dalian, China) was used for the second round of PCR. The primers used and the experimental procedure were described previously [28], [29]. The primer sequences are shown in Table 1. The tests used disposable supplies (pipette tips, gloves, etc), and were carried out by skilled personnel. Two negative controls were included for each experiment. The positive results were repeated twice.

**Table 1:** Primers used in this study.

Nested PCR	Name	Sequences(5′–3′)	Position (HXB2)
1st PCR	MAW-26	TGGAAATGTGGAAAGGAAGGAC	2147–2166
	RT-21	CTGTATTTCTGCTATTAAGTCTTTTGATGGG	2539–2519 R
2nd PCR	PRO-1	CAGAGCCAACAGCCCCACCA	2946–2961
	RT-20	CTGCCAGTTCTAGCTCTGCTTC	2050–2029 R
For sequencing	PRO-1	CAGAGCCAACAGCCCCACCA	3539–3509
	RTA	GTTGACTCAGATTGGTTGCAC	2147–2166
	RTB	CCTAGTATAAACAATGAGACAC	3462–3441

### Electrophoresis and Sequencing

For electrophoresis, 5 µl of first round PCR product was loaded onto a 2.0% agarose gel. The gel was run with a constant voltage of 120V for 25min. PCR positive products were used for sequencing analysis(SinoGenoMax Co, Ltd, Beijing). The amplification products were 1315-bp in length. All the gene sequences were uploaded to the Genebank.

### HIV-1 Natural Drug Resistance-Associated Mutation Analysis

MEGA4.02 software was used to analyze the drug resistance gene sequences. The phylogenetic tree was constructed using the Bootstrap Neighbor Joining method. Genotyping was conducted using BLAST tool provided by the U.S. Los Alamos HIV database (http://www.hiv.lanl.gov). The edited sequences were submitted to the Stanford HIV Drug Resistance Database (http://hivdb.stanford.edu/index.html) (Version 6.2.0, last updated 05/29/12) for analysis of drug resistance, resistance loci and resistance-associated mutations. The preliminary target sequences were evaluated for drug sensitivity, potentially low resistance, low resistance, medium resistance or high resistance.

## Results

### General

One hundred and seven HIV-1 pol gene sequences were obtained. The positive rate was 71.3% (107/150). The ages of patients were between 29 and 60, with an average age of 45.0. Among them, 66 (61.7%) were males, with an average age of 45.4 years, and 41 (38.3%) were females, with an average age of 44.6 years. As determined by Stanford HIV Drug Resistance Database, 40.2% (43/107) samples had one or more resistance-associated mutations. These patients had an average age of 47.6 years, with male accounting for 69.8% (30/43, mean age 47.7 years), and female accounting for 30.2% (13/43, mean age 47.5 years). The CD4 cell counts, plasma HIV RNA levels and HCV RNA levels were determined, and the results were summarized in the table 2. However, there was not enough blood plasma for HBV measurement.

**Table 2:** Characteristics of treatment-naive HIV-infected study population in former paid blood donors in Henan province, China.

Characteristics	Total n (%)	HIV-1 pol gene sequenceswere Obtained n (%)	HIV drug resistance n (%)
Sex	150	107	43
Male	86(57.3)	66(61.7)	30(69.8)
Female	64(42.7)	41(38.3)	13(30.2)
Median age at inclusion, years (range)	44.7 (29–60)	45.0(29–60)	47.6(30–60)
Baseline CD4^+^ T cell count (cells/ml)	73	54	21
≥500	7(9.6)	5(9.3)	0
350–499	38(52.1)	26(48.1)	12(57.1)
200–349	28(38.3)	23(42.6)	9(42.9)
50–199	0	0	0
<50	0	0	0
HIV Baseline viral load (copies/ml plasma)	150	107	43
<1000	2(1.3)	0	0
1000–9999	26(17.3)	3(2.8)	0
10 000–99 999	85(56.7)	67(62.6)	27(62.8)
>100 000	37(24.7)	37(34.6)	16(37.2)
HCV Baseline viral load (copies/ml plasma)	150	107	43
<1000	54(36.0)	39(36.5)	13(30.2)
1000–9999	3(2.0)	0	0
10 000–99 999	9(6.0)	3(2.8)	2(4.7)
>100 000	84(56.0)	65(60.7)	28(65.1)

### Phylogenetic Analysis of the Characteristics of HIV-1 Natural Resistance Mutations

**Figure 1:**
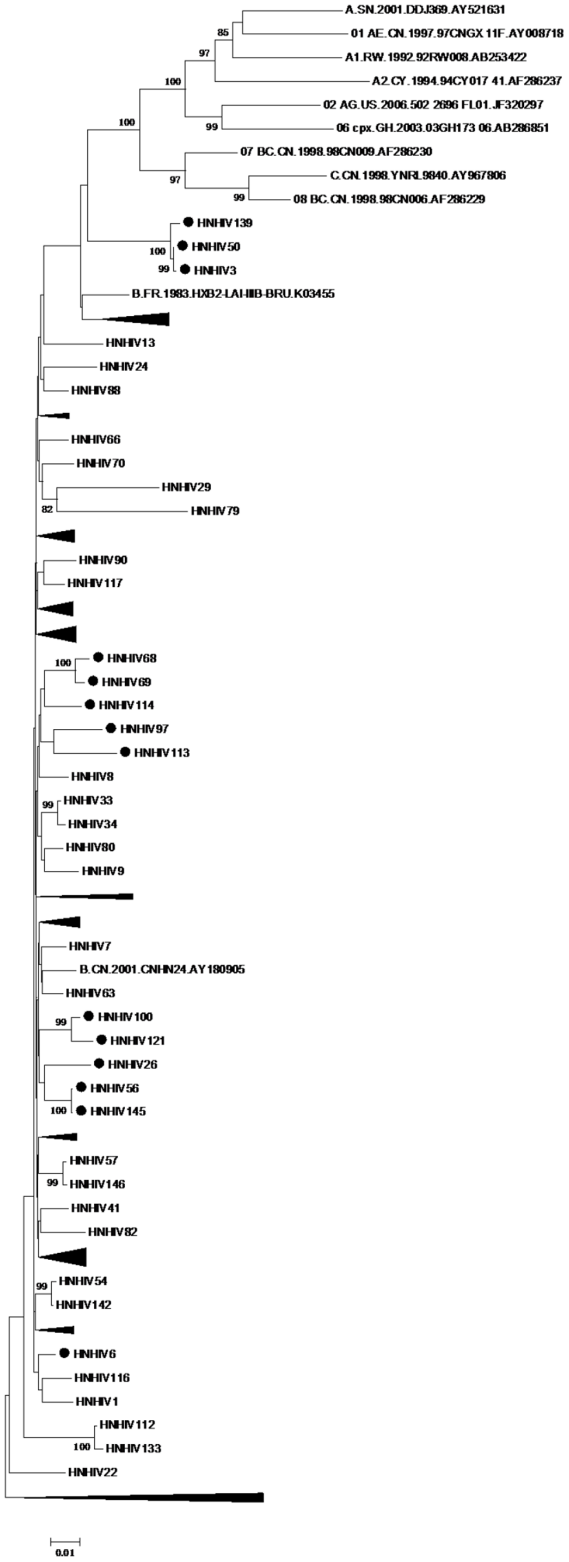
Phylogenetic analysis the characteristic of HIV-1 natural resistance mutations in former paid blood donors in Henan Province. The bootstrap values were performed by 5000, greater than 80 clusters shown in figure, black spots represent the drug-resistant mutations samples, black triangles represent the aggregation of the same cluster, reference strains for subtype B are: B.CN.2001.CNHN24.AY180905 and B.FR.1983.HXB2-LAI-IIIB-BRU.K03455.

As shown in Figure 1, the distribution of 107 sequences was scattered. They were mainly clustered together with reference strains B.CN.2001.CNHN24.AY180905 and B.FR.1983.HXB2-LAI-IIIB-BRU.K03455. The subtype of these strains was B subtype. Samples with black spots represented the drug-resistant mutations. HNHIV3, HNHIV50, and HNHIV139 were clustered together, and had L10I mutation. HNHIV46, HNHIV74, HNHIV77, and HNHIV137 were clustered together, with HNHIV46 and HNHIV137 having A71V, D67N, K101E, and G190A mutations, HNHIV74 having A71T mutation, and HNHIV77 having A71V and K103N mutations. Samples HNHIV6, HNHIV58, and HNHIV149 were clustered together, and had A71T mutation. Samples HNHIV19, HNHIV68, HNHIV69, HNHIV93, HNHIV97, HNHIV113, and HNHIV114 were clustered together, with HNHIV19 and HNHIV93 grouped in a smaller cluster. The drug resistance mutation loci included A71V, D67H, T69N, K70R, T215F, K219E, A98G, V179D, and Y181C, with HNHIV97 and HNHIV113 clustered together. HNHIV97 locus mutations included D67N, T69A, K70R, T215Y, K219E, K103N, and Y181C. HNHIV113 site mutations included A71T, M41L, M184V, L210W, T215Y, K103N, and Y181C. Samples HNHIV68, HNHIV69, and HNHIV114 were clustered together, with both HNHIV68 and HNHIV69 having mutations K103N and Y181C, HNHIV114 having mutation A71V. Samples HNHIV26, HNHIV56, and HNHIV145 were clustered together, with HNHIV26 having mutations L10I, A71T, K103N, and Y181C, and HNHIV56 and HNHIV145 having mutation A71T. Samples HNHIV4, HNHIV83, HNHIV100, and HNHIV121 were clustered together, and had A71T mutation. In addition, the PCR sequences with <1% difference are 51 and 140, 56 and 145.

In samples with L10I mutation, HNHIV24 was not clustered together with HNHIV3, HNHIV50 and HNHIV139. In samples with A71T mutation, HNHIV6, HNHIV58, and HNHIV149 were clustered together, HNHIV4, HNHIV83, HNHIV100, and HNHIV121 were clustered together, and HNHIV10 and HNHIV74 had scattered distribution. In samples with A71V mutation, HNHIV23, HNHIV44, HNHIV63, and HNHIV114 had scattered distribution. All samples with both A71V and K103N mutations had scattered distribution. In samples with the same mutation, HNHIV19 and HNHIV93 were clustered together, and HNHIV65 was not clustered together with HNHIV68 and HNHIV69.

### The Distribution of Natural Resistance Locus

PI had no major resistance-associated mutations. PI minor resistance-associated mutation rate was 30.8% (33/107). The mutation positions were A71T, L10I, A71V, and Q58E, with A71T and L10I happening simultaneously, leading to NFV potential low resistance. Q58E led to TPV/r potential low-level resistance, with a resistance rate of 1.9% (2/107).

NRTI resistance-associated mutations were found in 10 patients (9.3%). Nine were resistant to NRTI, with a resistance rate of 8.4% (9/107). In these cases, 4.7% (5/107) were high resistance, 1.9% (2/107) were intermediate resistance, 6.5% (7/107) were low resistance and 1.9% (2/107) were potential low resistance. Single point mutation M184V led to high resistance to 3TC and FTC, low drug resistance to ABC, and potential low resistance to DDI; T215Y caused intermediate resistance to AZT and D4T, and low resistance to ABC, DDI and TDF; M184L did not cause resistance to NRTI. When comparing HNHIV97 and HNHIV93, T215F was replaced by T215Y, which changed intermediate resistance to DDI to low resistance.

NNRTI resistance-associated mutations were found in 20 cases (18.7%). Eighteen cases were NRTI resistant, with a resistant rate of 16.8% (18/107). Among them, 15.9% (17/107) were high resistance, 10.3% (11/107) were intermediate resistance, 1.9% (2/107) were low resistance, and 0.9% (1/107) were potential low resistance. In seven cases Y181C occurred together with K103N, causing high resistance to EFV and NVP, and intermediate resistance to ETR and RPV. The mutations of both K103N and V108I caused high resistance to EFV and NVP. Y181C caused intermediate resistance to ETR and RPV. K101E and G190A caused high resistance to NVP, intermediate resistance to EFV, and low resistance to ETR and RPV. V179E did not cause resistance to NNRTI. Seven patients (6.5%) had PI minor, NRTI and NNRTI resistant mutants. Nine patients (8.4%) had both NKTI and NNRTI resistance mutations. Specimens HNHIV19 and HNHIV93 had mutations at the same locus, and HNHIV46 and HNHIV137 had mutations at the same locus. The results were summarized in the table 3.

**Table 3:** HIV-1 drug resistance mutations natural distribution of former paid blood donation population in Henan province.

Number	Gender	Age	Drug Resistance Interpretation: PR				Drug Resistance Interpretation: RT			
			PI Maj	SpeR	PI Min	Spe R	NRTI	Spe R	NNRTI	Spe R
HNHIV1	female	53	–	–	A71T	–	–	–	–	–
HNHIV3	male	51	–	–	L10I	–	–	–	–	–
HNHIV4	female	46	–	–	A71T	–	–	–	–	–
HNHIV6	female	36	–	–	A71T	–	–	–	–	–
HNHIV10	male	50	–	–	A71T	–	–	–	–	–
HNHIV13	female	37	–	–	A71V	–	M184V	**H**:3TC,FTC **L**:ABC **P**:DDI	K103N, Y181C	**H**:EFV,NVP **I**:ETR,RPV
HNHIV15	male	57	–	–	–	–	K70N	**P**:3TC,ABC,D4T,DDI,FTC,TDF	V108I	**P**:NVP
HNHIV19	female	49	–	–	A71V	–	D67H, T69N, K70R, T215F, K219E	**H**:AZT,D4T **I**:DDI,TDF **L**:ABC	A98G, V179D, Y181C	**H**:NVP **I**:EFV,ETR,RPV
HNHIV20	male	56	–	–	A71V	–	–	–	K103N	**H**:EFV,NVP
HNHIV23	male	46	–	–	A71V	–	–	–	–	–
HNHIV24	male	54	–	–	L10I	–	–	–	–	–
HNHIV26	male	49	–	–	L10I, A71T	**P**:NFV	–	–	K103N, Y181C	**H**:EFV,NVP **I**:ETR,RPV
HNHIV29	male	37	–	–	A71T	–	T215Y	**I**:AZT,D4T **L**:ABC,DDI,TDF	K103N	**H**:EFV,NVP
HNHIV36	male	51	–	–	Q58E	**P**:TPV/r	–	–	–	–
HNHIV39	male	49	–	–	A71V	–	–	–	K103N	**H**:EFV,NVP
HNHIV44	male	51	–	–	A71V	–	–	–	–	–
HNHIV46	male	47	–	–	A71V	–	D67N	**L**:AZT,D4T	K101E, G190A	**H**:NVP **I**:EFV **L**:ETR,RPV
HNHIV50	male	41	–	–	L10I	–	–	–	–	–
HNHIV56	male	49	–	–	A71T	–	–	–	–	–
HNHIV58	male	46	–	–	A71T	–	–	–	–	–
HNHIV63	male	46	–	–	A71V	–	–	–	–	–
HNHIV65	female	47	–	–	–	–	–	–	K103N, Y181C	**H**:EFV,NVP **I**:ETR,RPV
HNHIV68	male	52	–	–	–	–	–	–	K103N, Y181C	**H**:EFV,NVP **I**:ETR,RPV
Number	Gender	Age	Drug Resistance Interpretation: PR				Drug Resistance Interpretation: RT			
			PI Maj	SpeR	PI Min	Spe R	NRTI	Spe R	NNRTI	Spe R
HNHIV69	male	53	–	–	–	–	–	–	K103N, Y181C	**H**:EFV,NVP **I**:ETR,RPV
HNHIV70	male	60	–	–	–	–	–	–	K103N, V108I	**H**:EFV,NVP
HNHIV74	male	49	–	–	A71T	–	–	–	–	–
HNHIV76	male	58	–	–	–	–	–	–	K103N	**H**:EFV,NVP
HNHIV77	male	50	–	–	A71V	–	–	–	K103N	**H**:EFV,NVP
HNHIV79	female	54	–	–	–	–	M184L	–	–	–
HNHIV83	female	53	–	–	A71T	–	–	–	–	–
HNHIV86	male	36	–	–	–	–	–	–	V179E	–
HNHIV93	female	35	–	–	A71V	–	D67H, T69N, K70R, T215F, K219E	**H**:AZT,D4T **I**:DDI,TDF **L**:ABC	A98G, V179D, Y181C	**H**:NVP **I**:EFV,ETR,RPV
HNHIV97	male	56	–	–	–	–	D67N, T69A, K70R, T215Y, K219E	**H**:AZT,D4T **I**:TDF **L**:ABC,DDI	K103N, Y181C	**H**:EFV,NVP **I**:ETR,RPV
HNHIV100	female	47	–	–	A71T	–	–	–	–	–
HNHIV113	male	30	–	–	A71T	–	M41L, M184V, L210W, T215Y	**H**:3TC,ABC,AZT,D4T,DDI,FTC **I**:TDF	K103N, Y181C	**H**:EFV,NVP **I**:ETR,RPV
HNHIV114	female	39	–	–	A71V	–	–	–	–	–
HNHIV116	female	54	–	–	A71T	–	–	–	–	–
HNHIV121	male	36	–	–	A71T	–	–	–	–	–
HNHIV137	male	49	–	–	A71V	–	D67N	**L**:AZT,D4T	K101E, G190A	**H**:NVP **I**:EFV **L**:ETR,RPV
HNHIV139	male	51	–	–	L10I	–	–	–	–	–
HNHIV145	male	40	–	–	A71T	–	–	–	–	–
HNHIV149	female	52	–	–	A71T	–	–	–	–	–
HNHIV150	male	48	–	–	–	–	–	–	V179E	–

PI mar, PI Major Resistance Mutations; PI min, PI Minor Resistance Mutations; Spe R, Specific resistance; H, High-level resistance; I, Intermediate resistance; L, Low-level resistance; P, Potential low-level resistance; ATV/r, atazanavir/r; DRV/r, darunavir/r; FPV/r, fosamprenavir/r; IDV/r, indinavir/r; LPV/r, lopinavir/r; NFV, nelfinavir; SQV/r, saquinavir/r; TPV/r, tipranavir/r; 3TC, lamivudine; ABC, abacavir; AZT, zidovudine; D4T, stavudine; DDI, didanosine; FTC, emtricitabine; TDF, tenofovir; EFV, efavirenz; ETR, etravirine; NVP, nevirapine; RPV, rilpivirine; Mutation sites according to the area of the amino acid position in the form of so-called “M184V” records, said sharing sequence of RT’s 184th “M” was replaced by “V”.

### HIV Drug Resistance Mutation Annotations

In PI minor resistance-associated mutations, L10I accounted for 4.7%, which is slightly lower than the rate (5%–10%) of untreated population in the Stanford HIV drug resistance database annotation. A71T and A71V mutation rates were 14.9% and 11.2%, respectively. These were polymorphic mutations, and occurred at a higher rate than the rate (2–3%) of the untreated population in the database annotation.

In the RT region, among mutations associated with NRTI resistance, M41L often occurred together with T215Y, which caused high resistance to AZT and d4T, and low resistance to DDI, ABC and TDF. D67N accounted for 2.8% and caused AZT and d4T resistance. T69A and T69N accounted for 0.9% and 1.9%, respectively. Mutations at this site are usually caused by drug selection pressure. But the samples were collected before antiviral treatment in the present study. M184L was a rare mutation, which accounted for 0.9%. M184V accounted for 1.9%, and was responsible for high resistance to 3TC and FTC, and low-level resistance to ddI and ABC. L210W was often associated with M41L and T215Y mutations, and caused all NRTIs except 3TC and FTC resistance. K219E accounted for 2.8%, and could decrease AZT sensitivity in the presence of K70R or T215Y/F. Among mutations associated with NNRTI resistance, A98G accounted for 1.9%, and decreased the NVP and EFV sensitivity. K103N accounted for 12.1%, and caused high-level resistance to NVP and EFV. V108I accounted for 1.9%, and reduced NVP and EFV susceptibility. V179D/E reduced NVP and EFV susceptibility by about 2-fold. K103R and V179D mutations reduced NVP and EFV susceptibilities by about 10-fold. Y181C accounted for 8.4%, and caused high-level resistance to NVP. G190A accounted for 1.9%, and caused high level resistance to NVP and intermediate resistance to EFV. The results were summarized in the table 4.

**Table 4:** Resistance loci feature comments.

	Mutation	n	%	Comments form Stanford HIV Drug Resistance Database
PI Mar	–	–	–	–
PI Min	L10I	5	4.7	L10I/V occur in 5–10% of untreated persons.
	Q58E	1	0.9	Q58E associated with decreased susceptibility to TPV/r and possibly other PIs.
	A71T/V	16/12	14.9/11.2	A71T/V occur in 2–3% of untreated persons.
NRTI	M41L	1	0.9	M41L usually occurs with T215Y. confer high-level resistance to AZT and d4T and low level resistance to ddI, ABC, and TDF.
	D67H	2	1.9	D67H is a highly unusual mutation at this position.
	D67N	3	2.8	D67N contributes resistance to AZT and d4T.
	T69A/N	1/2	0.9/1.9	T69N/S/A/I are NRTI-selected mutations.
	K70N	1	0.9	K70Q/N/S/T are rare NRTI-selected mutations.
	K70R	3	2.8	K70R causes intermediate resistance to AZT and low-level resistance to d4T and TDF.
	M184L	1	0.9	M184L is a unusual mutation at this position.
	M184V	2	1.9	M184V/I cause high-level resistance to 3TC and FTC and low-level resistance to ddI and ABC.
	L210W	1	0.9	L210W contributes resistance to each of the NRTIs except 3TC and FTC. It usually occurs with the mutations M41L and T215Y.
	T215F/Y	2/3	1.9/2.8	T215F causes AZT and D4T resistance and reduces susceptibility to ABC, ddI, and TDF particularly in combination with M41L and L210W. T215F occurs more commonly (than T215Y) with Type II TAMs.
	K219E	3	2.8	K219Q/E decrease AZT and probably d4T susceptibility when present with K70R or T215Y/F.
NNRTI	A98G	2	1.9	A98G reduces NVP and EFV susceptibility by about 5-fold and 3-fold, respectively.
	K101E	2	1.9	K101E causes intermediate resistance to NVP and low-level resistance to EFV, ETR, and RPV.
	K103N	13	12.1	K103N causes high-level resistance to NVP, and EFV.
	V108I	2	1.9	V108I reduces NVP and EFV susceptibility.
	V179D/E	2/2	1.9/1.9	V179D/E occur in ∼1% of NNRTI-naive individuals and alone reduce NVP and EFV susceptibility.
	Y181C	9	8.4	Y181C causes high-level resistance to NVP, ∼2-fold decreased susceptibility to EFV, and ∼5-fold decreased susceptibility to ETR and RPV.
	G190A	2	1.9	G190A causes high level resistance to NVP and intermediate resistance to EFV.

### Natural Resistance Rate of HIV-1 in Former Paid Blood Donation Population

In the protease region, NFV and TPV/r potential resistance and low resistance rates were both 0.9%. In the RT region, NRTI resistance-associated mutations were mainly high resistance, with AZT and D4T high resistance rate of 3.7%. The next most often mutation was low resistance, with ABC low resistance rate of 4.7%. High resistance did not occur with TDF, but intermediate resistance rate was as high as 3.7%. Intermediate resistance and low resistance did not occur with 3TC and FTC. NRTI resistance rates were: 3TC 2.8% (3/107), ABC 6.5% (7/107), AZT6.5% (7/107), D4T 7.5% (8/107), DDI 6.5% (7/107), FTC 2.8% (3/107), and TDF5.6% (6/107). Overall NNRTI resistance was mainly high resistance, with EFV and NVP resistance rates of 10.3% and 14.0%, respectively. The next most often mutation was intermediate resistance, with ETR and RPV intermediate resistance rates both of 8.4%. Low resistance and potential low resistance did not occur with EFV. High resistance and potentially low resistance did not occur with ETR and RPV. Intermediate resistance and low resistance did not occur with NVP. NNRTI resistance rates were: EFV 14.0% (15/107), ETR 10.3% (11/107), NVP 15.4% (16/107), and RPV 10.3% (11/107) (Table 5). NNRTI resistance was the most serious, followed by NRTI resistance. PI resistance was the least serious.

**Table 5:** Natural resistance rate of HIV-1 in Henan former paid blood donation population.

		High-level resistance		Intermediate resistance		Low-level resistance		Potential low-level resistance	
	Drugs	n	%	n	%	n	%	n	%
Protease Inhibitors	ATV/r	–	–	–	–	–	–	–	–
	DRV/r	–	–	–	–	–	–	–	–
	FPV/r	–	–	–	–	–	–	–	–
	IDV/r	–	–	–	–	–	–	–	–
	LPV/r	–	–	–	–	–	–	–	–
	NFV	–	–	–	–	–	–	1	0.9
	SQV/r	–	–	–	–	–	–	–	–
	TPV/r	–	–	–	–	–	–	1	0.9
Nucleoside RTI	3TC	2	1.9	–	–	–	–	1	0.9
	ABC	1	0.9	–	–	5	4.7	1	0.9
	AZT	4	3.7	1	0.9	2	1.9	–	–
	D4T	4	3.7	1	0.9	2	1.9	1	0.9
	DDI	1	0.9	2	1.9	2	1.9	2	1.9
	FTC	2	1.9	–	–	–	–	1	0.9
	TDF	–	–	4	3.7	1	0.9	1	0.9
Non-Nucleoside RTI	EFV	11	10.3	4	3.7	–	–	–	–
	ETR	–	–	9	8.4	2	1.9	–	–
	NVP	15	14.0	–	–	–	–	1	0.9
	RPV	–	–	9	8.4	2	1.9	–	–

### The Characteristics of Other Mutations

Polymorphic mutations were mainly in the protease region. The sites with mutation rates higher than 60.0% included: L63A/P/S/T 89.7% (96/107), among which L63P was 70.1% (75/107); V77I 82.2% (88/107); I72E/M/K/T/V 80.4% (86/107), among which I72V was 63.6% (75/107); I93L 75.7%; E35D 72.9%. In addition, there were some other mutation sites: I62V 24.3%, T12A/K/P/S 19.6%, R41K 15.9%, N37D/S 11.2%, I64L/M/V 10.3%, R57K 10.3%.

The reverse transcriptase region also contained polymorphic mutations. The sites with mutation rates higher than 60.0% included: I135A/L/M/R/T/V 93.5% (100/107), among which I135V was 80.4% (86/107); T200A/E/I/P/V 89.7% (96/107), among which T200A was 81.3% (87/107); Q278E/K/N/T 88.8% (95/107), among which Q278E was 85.0% (91/107); S162C/Y 82.2% (88/107), among which S162C was 81.3% (87/107); K277R/S 66.4% (71/107), among which K277R was 64.5% (69/107). Other mutation sites included: I293V 45.8%, V245E/G/I/K/M/L/Q/T 28.0%, R211E/G/M/K 26.2%, F214I 14.0%, K122E 14.0%, A272G/P/S 13.1%, Q207E/H/K 11.2%.

## Discussion

HIV-1 reverse transcriptase lacks the proof-reading function. As a result, it will produce a base mismatch roughly every 10, 000–30, 000 nucleic acids during the synthesis. Results from the HIV-1 drug resistance mutation research by the International AIDS Society-USA (updated in March 2013) have revealed that PI resistance mutation sites are L10I, K20M, V32I, M36I, M46I/L, I47V/A, I50V, Q58E, A71V, G73S, V82A/F/T, I84V, L89V,L90M; NRTIs resistance mutations are M41L, A62V, K65R, D67N, K70E/R, Y115F, M184V/I, L210W, T215Y/F, K219Q/E; and nNRTIs resistance mutations are V90I, L100I, K101E/P, K103N/S, V106A/I/M, E138A/G, Y181C/I/V, Y188C/L, G190S/A, M230L [24].

In Henan Province, one of the earliest areas in China with free anti-AIDS treatment, drug resistance caused by long-term treatment has long been a big concern. Drug-resistant strains of HIV in the region have certain characteristics. This study is a cross-sectional survey carried out on the drug-resistant strains of HIV in Henan Province. there are 43 drug resistance patients is unknown, the actual rate of primary drug resistance all 150 patients may also differ. All of 107 HIV-1 pol gene sequences were subtype B. This is consistent with the fact that subtype B is the main strain in patients infected with HIV through blood transmission in rural areas of central China[30]–[33].

In the phylogenetic tree, the distribution of 107 gene sequences was scattered, with some drug-resistance strains grouped in the same cluster. But in the same cluster, resistance loci were not identical, with the same resistance locus appeared in different clusters. This finding indicates that the prevalence of subtype B drug-resistant strains may be the result of both self-replication and mutual interaction of viruses in former paid blood donors in Henan Province[32]–[33]. Some PCR sequences with <1% difference. This may be the epidemiologic linkage, or strains from the same source. Although we tried to obtain detailed information from all patients, the information was not always available, especially that related to virus spreading.

The primary resistance rate was 17.7%, which is similar to the rate (17.0%) of newly diagnosed individuals reported by Johnson et al [20], and higher than the rate (11.6%) reported by Novak et al [34], It is also higher than the rate in the United States (10%) [35]and the rate reported by Wang X, et al [36] and Liao L, et al [37] in China (2.7%–3.8%). However, this rate is lower than that in Canada (18.5% in 1999–2000, 27.6% in 2001)[38]–[39]. The rate of resistance-associated mutations was 40.2%, of which PI had no major resistance-associated mutations. The rate of resistance-associated mutations of PI minor was 30.8%, and the rate of mutations causing potential low resistant was 1.9%. This mutation rate is similar to the rate (1.3%) reported by Hamers et al [21], and lower than that (11.1%) reported by Li et al [40]. The rate of NRTI resistance-associated mutation was 10.3%, which is higher than the rates reported by Novak (7.8%) and Hamers (2.5%). The rate of NNRTI resistance-associated mutation was 18.7%, which is higher than the rates reported by Novak (3.0%) and Hamers (3.3%) [21], [32]. In addition, 6.5% of cases had resistance-associated mutations in PI, NRTI and NNRTI, and 8.4% had resistance-associated mutations in both NRTI and NNRTI. One possible explanation of this phenomenon is that the subtype B drug resistance is different from other subtypes, which is consistent with the view of Chilton et al [41]. NNRTI resistance was the most severe. The rates were in the following order: EFV 14.0% (15/107), ETR 10.3% (11/107), NVP 15.4% (16/107), and RPV 10.3% (11/107). NRTI resistance was the next, and the rates were in the following order: 3TC 2.8% (3/107), ABC 6.5% (7/107), AZT6.5% (7/107), d4T 7.5% (8/107), DDI 6.5% (7/107), FTC 2.8% (3/107), and TDF5.6% (6/107). PI drug resistance was the least severe, with the rates of both NFV and TPV/r resistance being 0.9%.

The main reasons for the higher rates in the drug resistance survey include: 1) People were infected with a single route of transmission; 2) The sample size is small; 2) Although the detailed interviews were carried out, it still does not rule out the possibility that some patients occasionally took antiviral drugs. complain of untreated together with treated for a long time. 4) A few patients had resistance strains. 5) The high resistance rates may partly result from primary infection by resistant strains, rather than the in vivo virus evolution. So transmitted antiretroviral drug resistance (TDR) in treatment-naive HIV-infected Individuals is very important, and needs further in-depth research [42].

Characteristics of resistance loci in this study were different from the annotations in the Stanford HIV drug resistance database. M41L often occurred together with T215Y mutation, and caused high resistance to AZT and d4T. T215Y replaced T215F, which changed intermediate resistance to DDI to low resistance. When K103N and V108I mutations happened together, they caused high resistance to EFV and NVP. Generally resistance loci reduce HIV adaptability. But researches revealed some exceptions. For example K103N and Y181C mutations almost have no influence on the strains, and Y181C mutation even confers the virus better replication capability compared to the wild-type strain. Once the resistant mutation survives, it will not disappear. Therefore the incidence of drug resistance exhibited an increasing trend [43].

The mutation rates in the protease and reverse transcriptase regions are higher than 60.0%. These results reveal that the frequency of HIV mutation is relatively high, and multiple quasispecies could exist in the same infected individual. When mutations happen in the key regions, a new type or subtype may be created. Although the mutation does not directly lead to the occurrence of drug resistance, it can reduce the drug-resistant genetic barrier. compared with wild type can be selected by resistance to faster, when the key drug target nucleotide mutation, may lead to a corresponding change in drug sensitivity [44].

There are several limitations in this study. Firstly, this is a cross-sectional retrospective survey of natural resistance epidemiological in former paid blood donors in Henan Province. There was not enough information associated with clinical treatment. As a result the resistance monitoring results might be biased, and could not trace the resistance of individuals. Therefore, it is necessary to conduct further follow-up studies for drug treatment effect. Secondly, because the sample storage time was too long, only about two third of samples could be successfully analyzed. Thirdly, because the characteristics (demographic, time, risk factor, epidemiological and/or geographic) of the population were insufficient, the naturally occurring mutations that clustered phylogenetically were not properly described. Fourthly, the high prevalence of resistance to NNRTI was not properly discussed regarding its main causes and consequences, such as treatment coverage of infected population, low genetic barrier of this drug class, popular use of NNRTI due to its low cost. Further research is needed on the adaptability and replication ability of the resistance loci. This may be achieved through follow-up observation of the variation trend and the influence of drug-resistance. Since the sample size in this study is small, in the future study, we will try to increase the sample size.

In conclusion, through studying HIV drug resistance loci and the characteristics of natural resistance in former paid blood donors in Henan Province, we understand better the relationship between resistance loci and resistance. Understanding the natural resistance situation before treatment is important for the elucidation of the regular pattern of drug resistance genes, the rational use of antiviral drugs, the development of effective anti-virus treatments, the selection of salvage therapy, and for providing guidance to minimize or delay drug resistance.
